# Th1/Th2 immune regulation and functional resilience in older adults following severe COVID-19: a prospective cohort study

**DOI:** 10.1007/s41999-025-01293-x

**Published:** 2025-08-31

**Authors:** Daniela Cataneo-Piña, Leslie Chávez-Galán, Ranferi Ocaña-Guzmán, José Alberto Ávila-Funes, Tamas Fulop, Ivette Buendia-Roldan

**Affiliations:** 1 Geriatrics and Palliative Care Clinic, Instituto Nacional de Enfermedades Respiratorias Ismael Cosío Villegas, 14080 Mexico City, Mexico; 2 Research Unit, Instituto Nacional de Enfermedades Respiratorias Ismael Cosío Villegas, 14080 Mexico City, Mexico; 3 Instituto Nacional de Ciencias Médicas y Nutrición Salvador Zubirán, Mexico City, Mexico; 4 Research Center on Aging, Graduate Program in Immunology, Faculty of Medicine and Health Sciences, University of Sherbrooke, Sherbrooke, QC Canada

**Keywords:** Frailty, Resilience, COVID-19, Th1/Th2 balance, Immunosenescence

## Abstract

**Background:**

Older adults recovering from severe COVID-19 exhibit heterogeneous trajectories, ranging from persistent frailty to full functional recovery. The biological mechanisms underpinning resilience in this population remain poorly defined. This study aimed to investigate the association between Th1/Th2 immune regulation and functional resilience, defined as improvement in frailty status, among older survivors of severe COVID-19.

**Methods:**

We conducted a prospective study at a tertiary respiratory center in Mexico. Twenty-four patients aged 65 or older with a history of severe COVID-19 were assessed at 4 and 12 months post-discharge. Frailty was evaluated using a validated phenotype adapted for the Mexican population. Peripheral blood mononuclear cells (PBMCs) were analyzed by flow cytometry to quantify CD4+ T cell subsets, cytokine production (IFN-γ, TNF, and IL-10), and the expression of T-bet, GATA-3, and TIM-3.

**Results:**

Thirteen participants showed improved frailty status over 12 months. Resilient patients exhibited a higher Th1/Th2 (T-bet/GATA-3) ratio at 4 months post-discharge and increased IFN-γ and TNF production at 12 months. TIM-3 expression on CD4+ cells and circulating levels were also elevated in the resilient group.

**Conclusions:**

A Th1-skewed immune profile early after recovery and sustained proinflammatory cytokine activity are associated with resilience in older adults following severe COVID-19. These findings offer insight into immune mechanisms that may support functional recovery in aging populations.

**Supplementary Information:**

The online version contains supplementary material available at 10.1007/s41999-025-01293-x.

## Key summary points

**Aim:**

To investigate the role of Th1-driven proinflammatory regulation in the resilience of older adults one year after severe COVID-19

**Findings:**

Resilient patients exhibited a Th1-skewed immune profile, higher levels of proinflammatory cytokines (IFN-γ, TNF), and increased expression of the regulatory molecule TIM-3 compared to non-resilient patients.

**Message:**

A regulated Th1-driven proinflammatory response appears crucial for achieving resilience and recovery from frailty in older adults after severe COVID-19.

**Supplementary Information:**

The online version contains supplementary material available at 10.1007/s41999-025-01293-x.

## Introduction

Adverse life events—including illness, isolation, and physiological stress—challenge an individual’s capacity to maintain or regain functional equilibrium. As we age, this adaptive capacity, known as allostasis, becomes progressively impaired [1]. The diminished physiological reserves and increased allostatic load—defined as the cumulative burden of chronic stress and physiological dysregulation across multiple systems—reduce the ability of older adults to recover effectively from acute stressors [2], consequently leading to maladaptive responses and prolonged recovery [3].

It is strongly associated with adverse outcomes in COVID-19 patients. Resilience, in the geriatric context, refers to the capacity to recover function following acute illness or stress.

The COVID-19 pandemic exposed older adults to an unprecedented global stressor. Although they experienced the highest mortality during the acute phase, recovery outcomes varied widely; while some older survivors developed or worsened frailty, others regained their functional baseline [4, 5]. This variability highlights the concept of resilience, defined here as the capacity to recover functionally following acute illness [6]. While traditionally viewed through psychosocial lenses, resilience also has biological underpinnings, particularly involving immune system regulation [7].

The immune response to SARS-CoV-2 is orchestrated in part by CD4+ T cells, with Th1 and Th2 subsets playing complementary roles [8, 9]. At the transcriptional level, Th1 and Th2 polarization is regulated by lineage-defining nuclear factors. T-bet (T-box transcription factor TBX21) is the master regulator of Th1 differentiation, promoting IFN-γ expression and Th1-mediated immunity [10]. In contrast, GATA-3 (GATA binding protein 3) governs Th2 lineage commitment and facilitates the production of IL-4, IL-5, and IL-13 [11]. Assessing the relative expression of T-bet and GATA-3 provides insights into the Th1/Th2 immune axis and its potential role in shaping recovery trajectories following severe infections. A balanced Th1/Th2 profile appears critical for effective immune recovery and long-term homeostasis [12].

Frailty is a multidimensional syndrome of reduced physiological reserve and increased vulnerability to stressors [13], and is a prevalent and impactful outcome among older COVID-19 survivors, linked with poor quality of life and increased mortality [14]. However, the immune characteristics of older adults who recover from frailty are not well understood. This study aimed to characterize the Th1/Th2 immune profile in older survivors of severe COVID-19, comparing those who demonstrated resilience to those with persistent frailty at 1 year post-discharge.

## Methods

### Study design and participants

We conducted a prospective cohort study at the National Institute of Respiratory Diseases (INER) in Mexico City. The protocol was approved by the Institutional Ethics Committee (C15-22), and all participants provided written informed consent. From August 2021 to August 2022, we consecutively recruited participants from the INER multidisciplinary post-COVID-19 follow-up program.

Eligible individuals were aged ≥ 65 years, had a documented history of severe COVID-19 according to National Institutes of Health (NIH) criteria [15], required hospitalization during the acute phase, and completed their first post-discharge evaluation 10–12 weeks after hospital discharge, followed by a subsequent evaluation 12 months later.

Patient selection was intentionally stratified by baseline frailty status to enable comparison of recovery trajectories between frail and non-frail individuals. An equal proportion from each frailty category was included, facilitating a balanced assessment of frailty dynamics within the geriatric cohort. The follow-up time points were chosen based on prior longitudinal COVID-19 recovery studies reporting a plateau in early functional recovery by approximately 3–4 months, coinciding with the emergence of post-acute sequelae and clinically relevant frailty transitions [16]. The 12-month interval was selected to capture longer-term trajectories and late recovery patterns, consistent with follow-up frameworks in critical illness and post-COVID-19 research.

Exclusion criteria included current systemic immunosuppressive therapy (e.g., chemotherapy, high-dose corticosteroids), active malignancy under treatment, uncontrolled autoimmune disease, or refusal to participate. Consecutive recruitment was implemented to reduce selection bias and reflect the real-world profile of older survivors of severe COVID-19 managed at our institution.

### Frailty assessment and resilience definition

Frailty was evaluated using a phenotype adapted for the Mexican population [17], based on Fried’s criteria: weight loss, weakness, exhaustion, slowness, and low activity (Table S1). Patients with ≥ 3 criteria present were considered frail; those with 1 or 2 criteria were defined pre-frail, and those with no criteria were considered as robust. Assessments were conducted at 4 and 12 months by trained geriatricians. Resilience was operationally defined as a transition to a less frail category over follow-up, i.e., from frail to pre-frail or fit, or from pre-frail to fit, consistent with prior studies on functional recovery in older adults with respiratory diseases [18].

### Peripheral mononuclear cells (PBMCs) collection and culture

The blood sample was collected 4 and 12 months post-acute COVID-19 in a tube with spray-coated EDTA (BD Vacutainer 367863, Franklin Lakes, NJ, USA). Plasma was collected and kept at − 20 °C until use; freezing–thawing processes were avoided. Peripheral blood mononuclear cells (PBMCs) were collected via a Ficoll density gradient (Lymphoprep Axis-Shield, Oslo, Norway); their viability was determined using the Trypan blue dye (Sigma–Aldrich, St. Louis, MO, USA) exclusion method.

1 × 106 PBMCs/well were cultured and maintained for 24 h, at 37 °C, in a humidified atmosphere containing 5% CO_2_ and stimulated with monoclonal antibody (mAb) anti-CD3 and CD28 (1 μg/mL, BD Pharmingen). PBMCs in media or with isotype control (1 µg/mL) were included. Brefeldin was added 5 h before the end of the culture (1000x, 1 μL/mL, BD GolgiPlug, USA).

### Flow cytometry

At the end of the culture, PBMCs were recovered and stained with mAb anti-CD3, CD2, CD4, CD8, CCR7, CD45RA, TNF, IFN-γ, IL-4, IL-10, TIM-3, T-bet, and to identify and characterized T cells subpopulations. More details of the mAb can be found in Table S2.

Cytokines T-bet and GATA-3 T were evaluated by intracellular staining. Briefly, mAbs against surface molecules (CD3, CD4, CD8, CCR7, CD45RA, T-bet and GATA3) were incubated with cells for 20 min at 4 °C and washed; posteriorly, the cell pellet was suspended in fixation/permeabilization solution (eBioscience, Beverly, MA, USA) at 4 °C, washed with permeabilization buffer (eBioscience), and then stained with intracellular molecules (TNF-α, IFN-γ, IL-4, IL-10, TIM-3) at 4 °C and analyzed by flow cytometry.

Data were acquired using a FACS Aria II flow cytometer (Becton Dickinson, San Jose, CA, USA). Trypan blue cell counting was used to exclude dead cells. Fluorescence minus one (FMO) control was employed to set the gates for specific immune cell subpopulations. At least 50,000 events per sample were acquired. Compensation setup and calculation of the frequency of specific cell subsets were made using Flow Jo (Flow Jo, LLC, Ashland, OR, USA).

### Statistical analyses

The Shapiro–Wilk normality test was used to evaluate the data distribution. Data are shown as median values and interquartile ranges (IQR). According to the number of samples and normality test, statistical analyses were performed using Kruskal–Wallis’s test with Dunnett’s post-test and Mann–Whitney *U* test. Demographic characteristics and comorbidities were described using frequencies and percentages for categorical variables and medians and interquartile ranges for continuous variables. Differences between groups were analyzed using univariate analysis, with Fisher’s exact test for categorical variables and the Mann–Whitney *U* test for continuous variables. Bivariate logistic regression models were performed in order to identify the correlates of recovery from frailty status. The Stata v13 program was used to perform this statistical analysis. *p *value < 0.05 was considered statistically significant. Graphs were plotted by GraphPad Prism Software (v10).

## Results

### Baseline characteristics

We enrolled 24 patients with a median age of 72.4 years, at the beginning of the study, six were considered pre-frail, while eighteen were frail. After 12 months of follow-up, 13 patients improved their frailty status, with 4 in the pre-frail group and 9 in the frail group (Fig S1). Therefore, in the resilient group, we had 13 patients, and 11 were non-resilient. The sex distribution in the overall cohort was 25% female (*n* = 6). Among resilient participants (*n* = 13), 1 was female (7.7%), whereas in the non-resilient group (*n* = 11), 5 were female (45.5%). Most required mechanical ventilatory support during their hospital stay, with a median duration of 10.5 days. Common comorbidities included hypertension, diabetes mellitus, and ischemic heart disease, as shown in Table 1.

**Table 1:** Demographic characteristics, COVID-19 severity markers, and comorbidities are categorized according to improvement in frailty status at 1 year of follow-up

	Total (*n* = 24)	Resilient (*n* = 13)	Non-resilient (*n* = 11)	*p* value
Demographic characteristics				
Female sex (%)	**6** (**25**)	**1** (**8**)	**5** (**46**)	**0**.**01**
Marital status married (%)	8 (33)	4 (31)	4 (36)	0.5
Age (IQR)	72 (7–79)	72 (69–85)	71 (7–79)	0.3
Full immunization schedule against SARS-CoV-2 (%)	14 (58)	8 (62)	6 (55)	0.4
COVID-19 severity markers				
Invasive mechanical ventilation (%)	15 (63)	8 (62)	7(64)	0.9
IMV days (IQR)	10.5 (0–17)	13 (0–17)	9 (0–16)	0.6
Days of in-hospital stay (IQR)	18 (10–27)	20 (10–27)	12 (10–21)	0.5
Tracheostomy (%)	1 (4)	1 (8)	0 (0)	0.3
Gastrostomy (%)	1 (4)	1 (8)	0 (0)	0.3
Comorbidities				
Diabetes mellitus (%)	12 (50)	8 (62)	4 (36)	0.2
Systemic arterial hypertension (%)	19 (79)	12 (92)	7 (64)	0.08
Ischemic heart disease (%)	5 (21)	3 (23)	2 (18)	0.7
Chronic obstructive pulmonary disease (%)	4 (17)	2 (15)	2 (18.2)	0.8
Obstructive sleep apnea syndrome (%)	1 (4)	1 (8)	0 (0)	0.3
Hypothyroidism (%)	4 (17)	1 (10)	3 (19)	0.4
Acid-peptic disease (%)	6 (25)	3 (23)	3 (27)	0.8
Interventions provided				
Use of oral steroids (%)	12 (50)	7 (53.84)	5 (45.45)	0.671
Attendance to rehabilitation sessions (%)	21 (87.5)	12 (92.3)	9 (81.8)	0.064
Usual exercise at home (%)	16 (66.6)	11 (84.6)	5 (41.7)	0.054
Exercise at home 3 or more times a week (%)	**15** (**62**.**5**)	**11** (**84**.**6**)	**4** (**36**.**4**)	**0**.**02**
Exercise at home 30 or more minutes per day (%)	**10** (**41**.**6**)	**8** (**61**.**5**)	**2** (**18.2**)	**0**.**034**
Use of oral steroids (%)	12 (50)	7 (53.84)	5 (45.45)	0.671

The chi-squared test was used to compare differences in categorical variables between resilient and non-resilient patients

Mann–Whitney *U* test was employed to compare differences between medians and interquartile ranges

*IMV* invasive mechanical ventilation, *IQR* interquartile range

*p value* < 0.05 is considered significant

### Post-COVID program interventions

Patients were selected from a post-COVID clinic, where they received comprehensive care involving physical rehabilitation, medical treatment for multiple organ complications, and psychological and nutritional support. Among the recruited patients, half of them (*n* = 12; 50%) underwent oral steroid therapy to address lung inflammation. Nearly all patients (*n* = 21; 87.5%) participated in rehabilitation sessions, but only two-thirds continued with home-based exercise routines. Of those continuing home exercises, more than half (62.5%) exercised three or more times per week while about two-fifths (41.6%) exercised for at least thirty minutes on more than two occasions each week. The bivariate logistic regression analyses showed that engaging in at least 30 min of daily home exercise (OR 6.66, *p* 0.045) was associated with recovery from frailty status.

### CD4+ T cells subtypes were similar despite resiliency

**Fig. 1:**
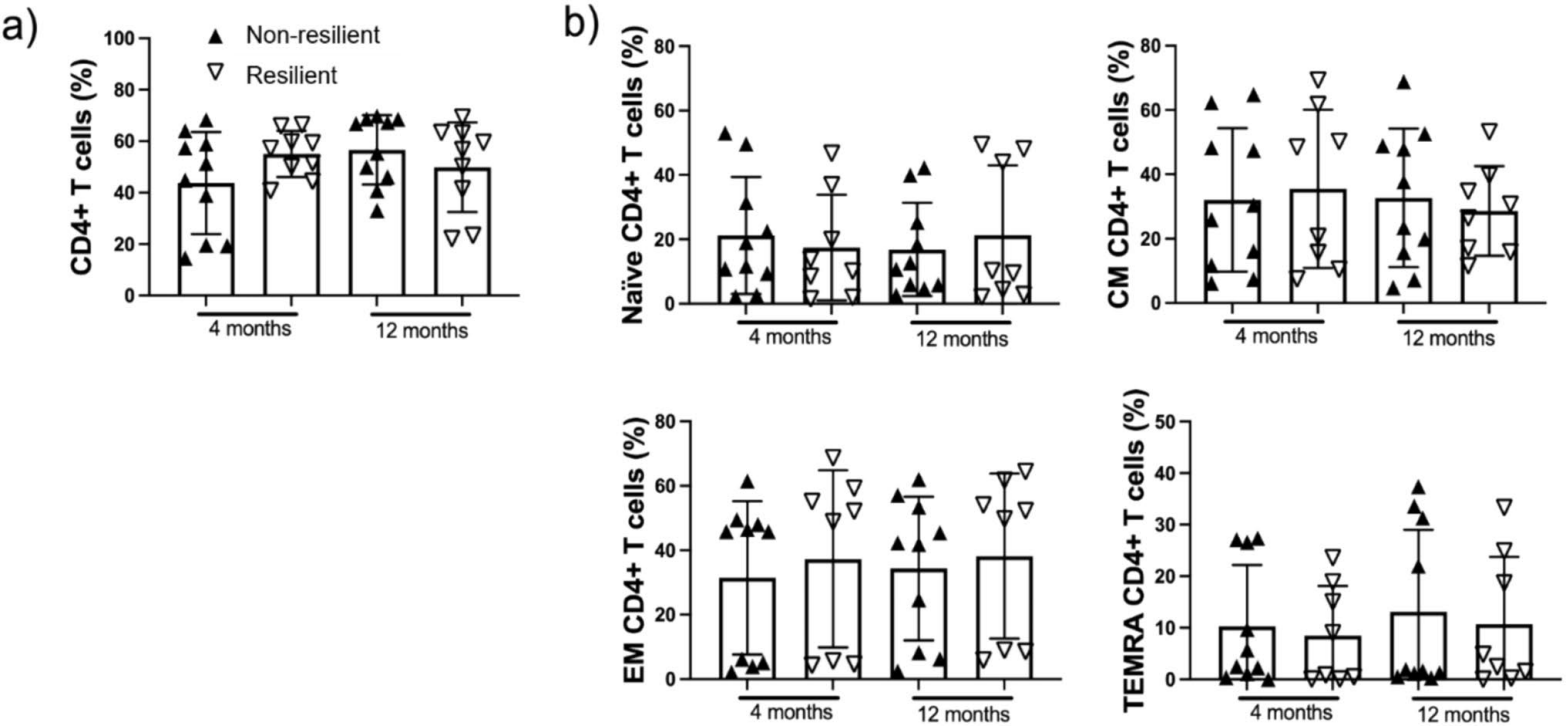
CD4+ T cell subsets are not affected in patients with frailty. Peripheral mononuclear cells from resilient and non-resilient patients were obtained after 4 and 12 months post-acute COVID-19 and prepared for flow cytometry. Based on the CCR7 and CD45RA expression, naïve, central memory (CM), effector memory (EM), and CD4 effector memory T cells re-expressing CD45RA (TEMRA) subsets were identified. Frequency of total CD4+ T cells (**a**). Frequency of naïve, CM, EM, and TEMRA CD4+ T cells (**b**). Data are represented as median and IQR values; each symbol represents an individual patient

The frequencies and proportions of CD4+ T cells were assessed by flow cytometry. The percentages and absolute counts of CD4+ T cells were comparable between individuals who recovered from their frailty status and those who did not. The CD4+ T cell subsets were classified as Naïve, T central memory (CM), T effector memory (EM), and terminally differentiated CD45RA-expressing memory (TEMRA) cells based on the expression of CCR7 and CD45RA. No significant differences were observed in these four CD4+ T cell subpopulations according to the patients’ resilience status (Fig. 1).

### Increased Th1 cells in resilient patients at 4 months from discharge

**Fig. 2:**
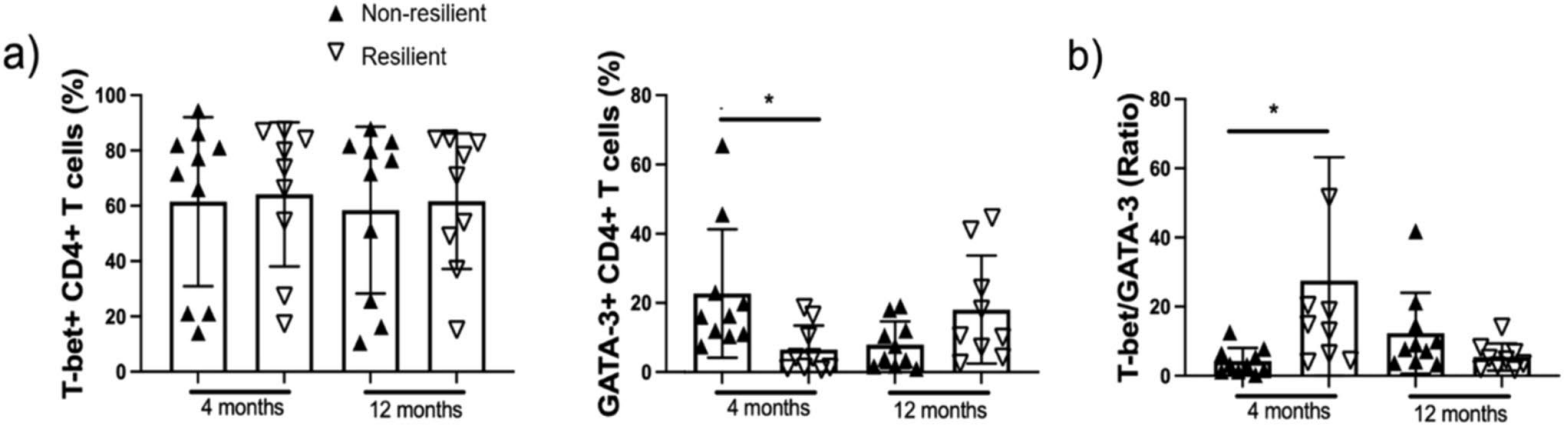
Non-resilient patients have decreased the Th1/Th2 ratio after 4 months post-acute COVID-19 compared to resilient. Peripheral mononuclear cells from non-resilient and resilient patients were obtained after 4 and 12 months post-acute COVID-19, and they were stimulated for 24 h with anti-CD3/CD28 (1 µg/mL) and prepared for flow cytometry. An unstimulated condition and isotype control were included as control stimulation. The T-bet and GATA3 expression was assessed in total CD4+ T cells (**a**). The T-bet/GATA3+ ratio was calculated and reported (**b**). Data are represented as median and IQR values; each symbol represents an individual patient. The Kruskal–Wallis test performed statistical comparisons, *p ≤ 0.05

At the initial assessment, patients who did not recover from frailty had a higher proportion of GATA-3-expressing CD4+ Th2 cells (*p* 0.05) (Fig. 2a), confirmed by higher T-Bet/GATA-3 ratio in resilient patients. This indicates a balance toward Th1 in resilient patients 4 months after discharge from COVID-19 (*p* < 0.05). No differences between resilient and non-resilient patients were observed in the T-bet or GATA-3 proportions at the 12-month assessment.

### Resilient patients exhibit higher proinflammatory cytokines at 12 months

**Fig. 3:**
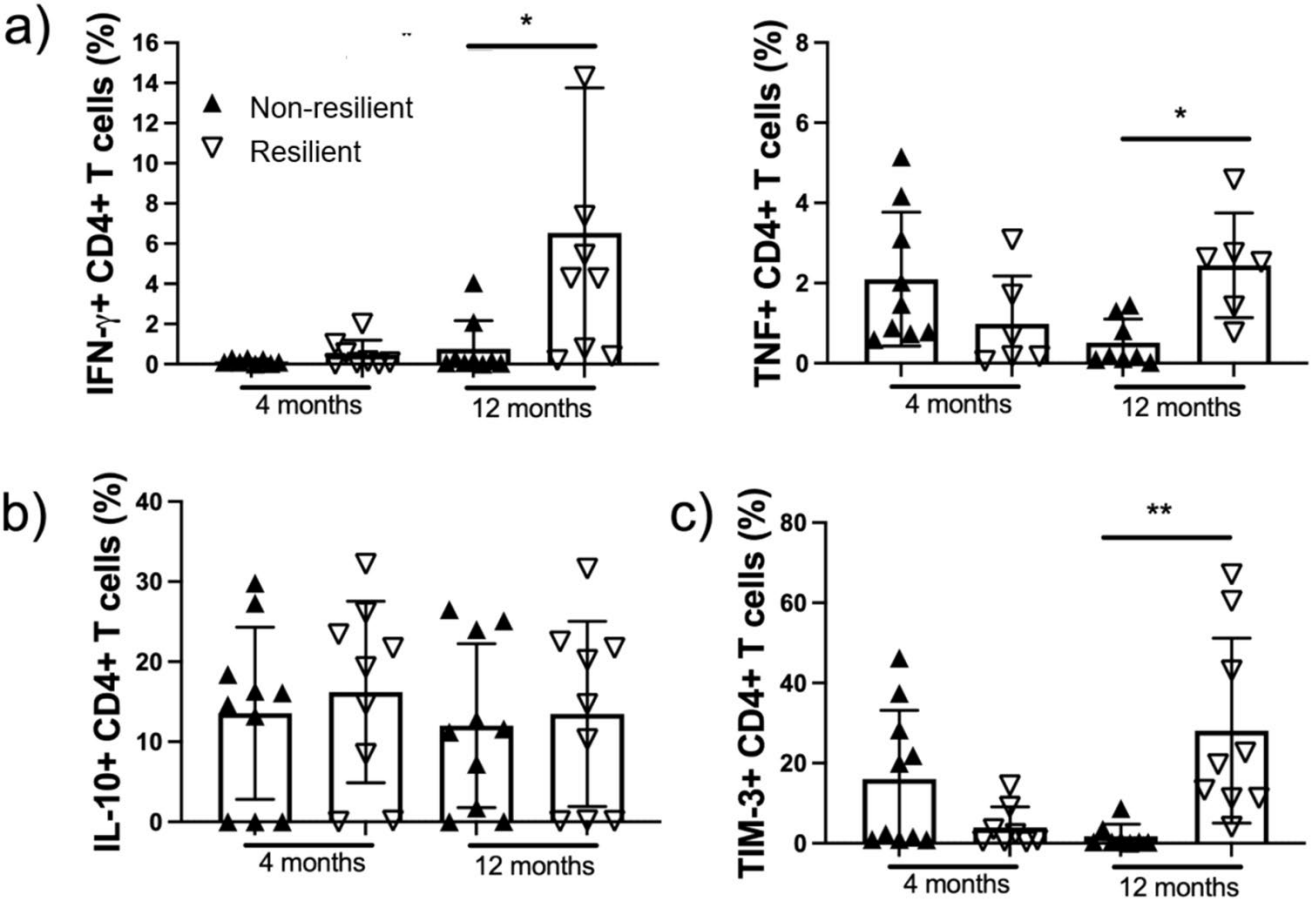
CD4+ T cells from non-resilient patients are inefficient in producing cytokines even after 12 months post-acute COVID. Peripheral mononuclear cells from non-resilient and resilient patients were obtained after 4 and 12 months post-acute COVID-19, and they were stimulated for 24 h with anti-CD3/CD28 (1 µg/mL) and prepared for flow cytometry. An unstimulated condition and isotype control were included as control stimulation. The frequency of CD4+ T cells producer of pro-inflammatory cytokines IFN-g and TNF was evaluated (**a**). The frequency of CD4+ T cells produced by the anti-inflammatory cytokine IL-10 was assessed (**b**). The frequency of CD4+ T cells expressing the regulatory molecule TIM-3 was evaluated (**c**). Data are represented as median and IQR values; each symbol represents an individual patient. The Kruskal–Wallis test performed statistical comparisons, **p 0.01, *p < 0.05

**Fig. 4:**
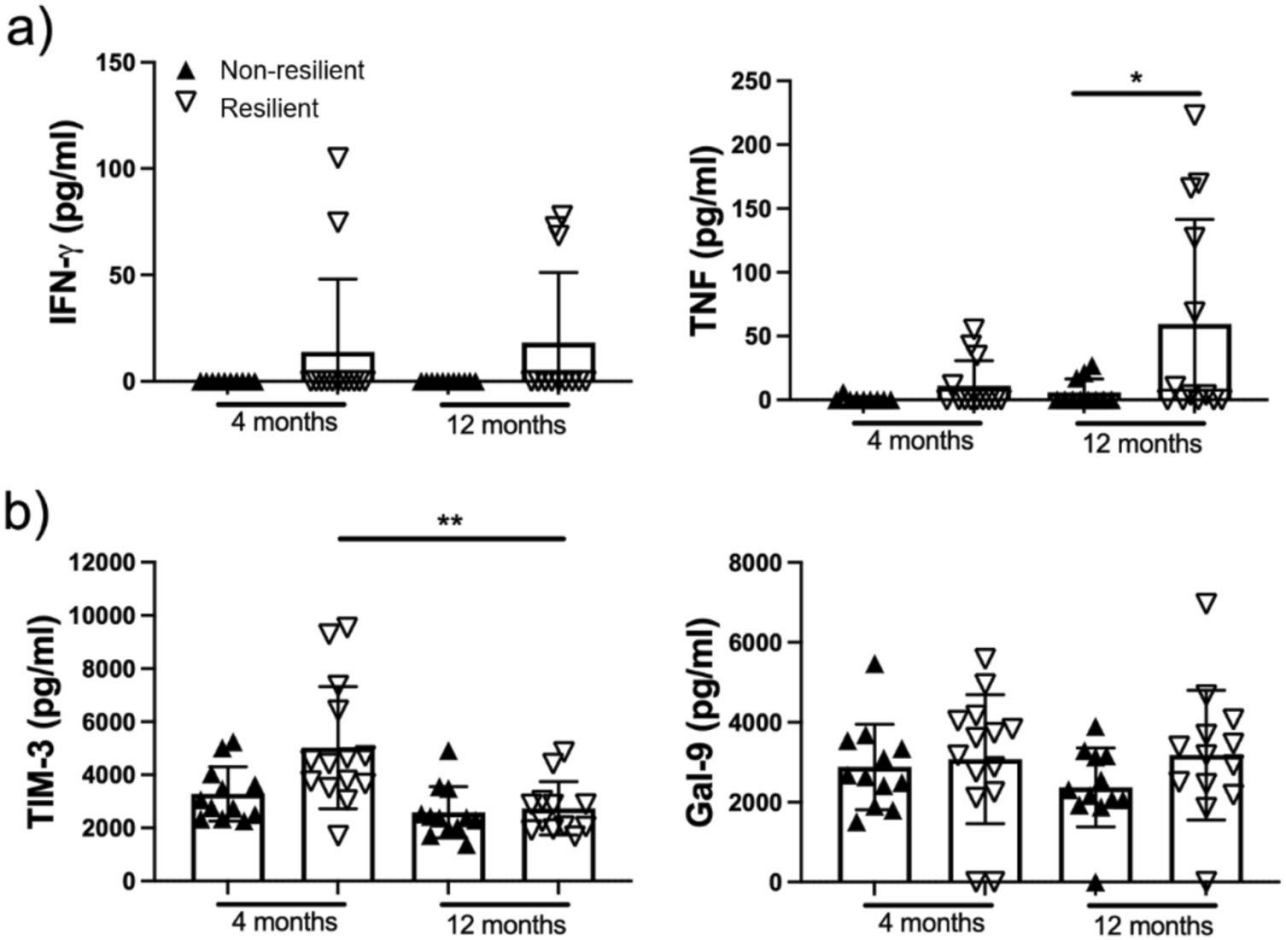
Resilient patients showed an increased systemic TNF and decreased TIM-3 levels after 12 months post-acute COVID-19. Plasma from non-resilient and resilient patients was obtained after 4 and 12 months post-acute COVID-19. Plasmatic levels of proinflammatory cytokines IFN-g and TNF were evaluated by ELISA (**a**). Plasmatic levels of the TIM-3 and its ligand Gal-9, molecules associated with regulation, were assessed by ELISA (**b**). Data are represented as median and IQR values; each symbol represents an individual patient. The Kruskal–Wallis test performed statistical comparisons, **p < 0.01, *p < 0.05

At the 4-month assessment, the proportions of positive CD4+ cells for IFN-γ, TNF, and IL-10 were similar between resilient and non-resilient patients. In contrast, at the 1-year assessment, resilient patients showed a higher proportion of CD4+ cells positive for the proinflammatory cytokines IFN-γ and TNF (*p* < 0.05) (Fig. 3a). Additionally, resilient patients had higher circulating levels of TNF at the 12-month assessment (*p* < 0.05) (Fig. 4a).

### Resilient patients have a higher TIM-3 expression

The expression of the TIM-3 molecule in CD4+ T cells was elevated in resilient patients at the 1-year assessment (*p* < 0.05) (Fig. 3c). Additionally, circulating levels of TIM-3 were higher in resilient patients 1 year after discharge (*p* < 0.05) (Fig. 4b).

## Discussion

This study explored how Th1 and Th2 immune regulation influences functional resilience in older adults recovering from severe COVID-19. The results offer novel insights into immunological pathways associated with recovery from frailty, providing a foundation for potential clinical applications.

Although aging is often described as a stochastic accumulation of physiological damage, it is increasingly recognized as a heterogeneous process shaped by lifelong exposures to environmental and inflammatory stressors [19, 20]. The immune system, with its complexity and adaptability, plays a central role in mediating this aging trajectory [21]. Investigating how immune regulation relates to the frailty phenotype offers valuable insights into why some older adults recover more effectively from acute illness—a phenomenon encapsulated by the concept of resilience.

COVID-19 has had profound effects on older individuals, contributing to declines in physical function, emotional well-being, and overall health [22]. These outcomes are strongly tied to immunosenescence and chronic low-grade inflammation, which impair recovery capacity [23]. Our findings highlight that immune features, particularly Th1/Th2 dynamics, help distinguish those who recover from frailty from those who remain impaired.

A key observation in our study was the elevated proportion of Th1 cells and a higher T-bet/GATA-3 ratio in resilient individuals at 4 months post-discharge. This shift toward a Th1-dominant immune profile is consistent with prior findings linking Th1 responses to improved viral clearance and better clinical outcomes in COVID-19 [24]. These results also support the emerging concept of immunological resilience, described by Ahuja et al. as the ability to maintain or restore immune competence and regulate inflammation during and after acute infection [25].

Conversely, a higher prevalence of GATA-3-expressing Th2 cells was associated with persistent frailty, in line with prior reports linking Th2-skewed immunity to poorer outcomes during the acute COVID-19 phase [7, 26]. Notably, 1 year after illness, resilient individuals had increased levels of IFN-γ and TNF, suggesting that sustained Th1 cytokine activity may support tissue repair and immune surveillance. These results challenge the conventional interpretation of chronic inflammation as inherently deleterious in aging, proposing instead a model of adaptive immune remodeling [27, 28].

Interestingly, our results also challenge the expectation that greater acute illness severity invariably predicts poorer long-term outcomes in older adults. In our cohort, some individuals who had pronounced inflammatory responses during acute COVID-19 subsequently demonstrated better functional recovery, accompanied by sustained Th1 cytokine activity and regulatory TIM-3 expression. This pattern may represent a form of adaptive immune remodeling, in which transiently heightened immune activation facilitates pathogen clearance and tissue repair, followed by regulatory mechanisms that prevent progression to chronic inflammation [29]. While the prevailing concept of inflamm-aging links heightened inflammation to adverse outcomes, our findings suggest that, in certain contexts, a controlled and well-resolved pro-inflammatory response may confer resilience. Similar observations have been reported in other post-critical illness populations, where balanced immune reactivation was associated with improved rehabilitation trajectories [30]. This paradox warrants further investigation, particularly to disentangle the interplay between immune activation, resolution pathways, and rehabilitation interventions.

Another important finding was the higher expression of TIM-3 on CD4+ T cells and in plasma among resilient patients at 1 year. Given that TIM-3 modulates T cell exhaustion and resolves inflammation [31, 32], this upregulation may represent a homeostatic mechanism that balances Th1-driven activation with immune regulation. Such duality—pro-inflammatory priming alongside regulatory restraint—may be critical in achieving recovery without tipping into chronic inflammation.

In our cohort, women were underrepresented among resilient participants, an observation that diverges from some aging and longevity literature [33] but is consistent with reports that, following major stressors, women can exhibit higher levels of stress-related symptoms and lower resilience scores than men. Notably, Lowe et al. showed that apparent sex differences in resilience may be partly explained by unequal background stressors (e.g., caregiving load, occupational exposures), which inflate women’s reported post-stress distress; once such stressors are considered, sex gaps narrow substantially [34]. Furthermore, the genetic architecture of resilience may differ by sex; a twin study by Boardman et al. [35] found sex differences in the heritability of resilience, suggesting both biological and socio-structural contributions. Together, these data provide plausible explanations for our finding—namely, that both contextual stressor burden and sex-linked biological factors could have constrained recovery opportunities for women in our setting. Although our sample was not powered for sex-stratified analyses, these patterns underscore the need for future work that explicitly measures background stressors and tests sex as an effect modifier in resilience models.

Beyond the immunological findings, our study incorporated a personalized rehabilitation component, revealing that both regular home-based exercise and a Th1-skewed immune profile were associated with frailty recovery. Participants engaging in consistent exercise (≥ 30 min, three times per week) showed greater improvements in frailty status, consistent with previous reports that low- to moderate-intensity exercise enhances physical function, mitigates sarcopenia, and improves quality of life in older post-COVID-19 patients [36]. Physical activity may also recalibrate immune balance, acting as a modulator of both innate and adaptive immunity through mechanisms that include increased production of anti-inflammatory cytokines and shifts in T helper cell polarization. Zamani et al. demonstrated that moderate exercise over 2 months increased the IFN-γ/IL-4 ratio [37], indicating a shift toward Th1 dominance. In our cohort, exercising participants similarly exhibited higher IFN-γ and TNF levels alongside better post-COVID pulmonary recovery [38]. While causality cannot be established, existing evidence suggests that exercise promotes Th1-type responses by enhancing IFN-γ production, modulating the naïve-to-memory T cell ratio, and attenuating chronic low-grade inflammation [39, 40]. These exercise-induced immunological adaptations, together with improvements in muscle strength and endurance, may act synergistically to reduce frailty risk and enhance resilience to future stressors, although the relative contributions of each remain to be determined.

From a clinical perspective, measuring immunological markers, such as Th1/Th2 ratios, TIM-3 expression, and cytokine profiles, could aid in stratifying older adults based on their risk of persistent frailty. This stratification may help guide post-discharge management, such as prioritizing high-risk individuals for early rehabilitation or immune-modulating interventions. These findings also support the development of novel therapeutic strategies aimed at recalibrating immune responses to promote functional recovery and resilience.

This study presents several strengths that enhance its scientific and clinical value. Its prospective design and 12-month follow-up allowed for longitudinal assessment of frailty recovery in older COVID-19 survivors. A major strength lies in the integration of clinical, functional, and immunological data, offering a multidimensional approach to understanding resilience. The use of flow cytometry to quantify intracellular markers, such as T-bet and GATA-3, provided mechanistic insights into Th1/Th2 regulation. Additionally, incorporating real-world data on rehabilitation and home-based exercise increased the clinical applicability of the findings. These features position the study at the intersection of geriatric and immunological research.

However, limitations include a small sample size and single-center design, which may affect generalizability. The sex distribution was unbalanced, with fewer women in the resilient group, which may have limited our ability to detect sex-specific recovery patterns. This imbalance likely reflects the real-world composition of older post-COVID-19 populations rather than recruitment bias, as participants were consecutively enrolled and stratified at baseline by frailty status. These findings should therefore be interpreted as hypothesis-generating and confirmed in larger, more balanced cohorts. The observational nature of the study also limits causal inference. Future research should involve larger, more diverse samples and investigate how Th1/Th2 polarization and TIM-3 expression predict recovery, ideally through longitudinal and interventional designs.

## Conclusion

Our study highlights the crucial role of Th1-skewed immune responses, sustained pro-inflammatory activity, and TIM-3-mediated regulation in promoting resilience among older adults recovering from severe COVID-19. Our findings suggest that a balanced Th1/Th2 immune profile, supported by physical activity and regulatory immune mechanisms, may be essential for reversing frailty and achieving favorable long-term outcomes. Identifying these immune signatures could guide the development of targeted interventions and inform personalized recovery pathways in geriatric populations post-COVID-19.

## Supplementary Information

Below is the link to the electronic supplementary material.

Supplementary file1 (DOCX 37 KB)

## Data Availability

The datasets generated and/or analyzed during the current study are available from the corresponding author upon reasonable request.

## References

[1] Kallen V, Tahir M, Bedard A, Bongers B, van Riel N, van Meeteren N (2021) Aging and allostasis: using Bayesian network analytics to explore and evaluate allostatic markers in the context of aging. Diagnostics (Basel) 11(2):157. 10.3390/diagnostics11020157 33494482 10.3390/diagnostics11020157 PMC7912325

[2] Sterling P, Eyer J (1988) Allostasis: a new paradigm to explain arousal pathology. In: Fisher S, Reason J (eds) Handbook of life stress, cognition and health. Wiley, New York, pp 629–649

[3] Whitson HE, Duan-Porter W, Schmader KE, Morey MC, Cohen HJ, Colón-Emeric CS (2016) Physical resilience in older adults: systematic review and development of an emerging construct. J Gerontol A Biol Sci Med Sci 71(4):489–495. 10.1093/gerona/glv202 26718984 10.1093/gerona/glv202 PMC5014191

[4] Hewitt J, Carter B, Vilches-Moraga A et al (2020) The effect of frailty on survival in patients with COVID-19 (COPE): a multicentre, European, observational cohort study. Lancet Public Health 5(8):e444–e451. 10.1016/S2468-2667(20)30146-8 32619408 10.1016/S2468-2667(20)30146-8 PMC7326416

[5] Owen RK, Conroy SP, Taub N et al (2021) Comparing associations between frailty and mortality in hospitalised older adults with or without COVID-19 infection: a retrospective observational study. Age Ageing 50(2):307–316. 10.1093/ageing/afaa167 32678866 10.1093/ageing/afaa167 PMC7454252

[6] Lima GS, Figueira ALG, Carvalho EC, Kusumota L, Caldeira S (2023) Resilience in older people: a concept analysis. Healthcare 11(18):2491. 10.3390/healthcare11182491 37761688 10.3390/healthcare11182491 PMC10531380

[7] Lee GC, Singh P, Apple CG, Ho YL, Zhang B, Goel R et al (2021) Immunologic resilience and COVID-19 survival advantage. J Allergy Clin Immunol. 10.1016/j.jaci.2021.08.021 34508765 10.1016/j.jaci.2021.08.021 PMC8425719

[8] Luckheeram RV, Zhou R, Verma AD, Xia B (2012) CD4+ T cells: differentiation and functions. Clin Dev Immunol 2012:925135. 10.1155/2012/925135 22474485 10.1155/2012/925135 PMC3312336

[9] Romagnani S (2000) T-cell subsets (Th1 versus Th2). Ann Allergy Asthma Immunol 85(1):9–21. 10.1016/S1081-1206(10)62426-X 10923599 10.1016/S1081-1206(10)62426-X

[10] Szabo SJ, Kim ST, Costa GL, Zhang X, Fathman CG, Glimcher LH (2000) A novel transcription factor, T-bet, directs Th1 lineage commitment. Cell 100(6):655–669. 10.1016/S0092-8674(00)80702-3 10761931 10.1016/s0092-8674(00)80702-3

[11] Zheng W, Flavell RA (1997) The transcription factor GATA-3 is necessary and sufficient for Th2 cytokine gene expression in CD4 T cells. Cell 89(4):587–596. 10.1016/S0092-8674(00)80240-8 9160750 10.1016/s0092-8674(00)80240-8

[12] Saravia J, Chapman NM, Chi H (2019) Helper T cell differentiation. Cell Mol Immunol 16(7):634–643. 10.1038/s41423-019-0223-5 30867582 10.1038/s41423-019-0220-6 PMC6804569

[13] Nicholson C, McCarthy M (2012) Frailty in primary care: a review of its conceptualization and implications for practice. BMC Med 10:4. 10.1186/1741-7015-10-4 22236397 10.1186/1741-7015-10-4 PMC3271962

[14] Marengoni A, Zucchelli A, Vetrano DL et al (2021) Beyond chronological age: frailty and multimorbidity predict in-hospital mortality in patients with coronavirus disease 2019. J Gerontol A Biol Sci Med Sci 76(3):e38–e45. 10.1093/gerona/glaa291 33216846 10.1093/gerona/glaa291 PMC7717138

[15] National Institutes of Health. Clinical spectrum of SARS-CoV-2 infection. [Internet]. Available from: https://www.covid19treatmentguidelines.nih.gov/overview/clinical-spectrum/. Accessed May 2025

[16] Morin L, Savale L, Pham T et al (2021) Four-month clinical status of a cohort of patients after hospitalization for COVID-19. JAMA 325(15):1525–1534. 10.1001/jama.2021.3331 33729425 10.1001/jama.2021.3331 PMC7970386

[17] Ávila-Funes JA, Amieva H, Barberger-Gateau P et al (2009) Cognitive impairment improves the predictive validity of the phenotype of frailty for adverse health outcomes: the three-city study. J Am Geriatr Soc 57(3):453–461. 10.1111/j.1532-5415.2008.02136.x 19245415 10.1111/j.1532-5415.2008.02136.x

[18] Maddocks M, Kon SS, Canavan JL et al (2016) Physical frailty and pulmonary rehabilitation in COPD: a prospective cohort study. Thorax 71(11):988–995. 10.1136/thoraxjnl-2016-208460 27293209 10.1136/thoraxjnl-2016-208460 PMC5099190

[19] López-Otín C, Blasco MA, Partridge L, Serrano M, Kroemer G (2013) The hallmarks of aging. Cell 153(6):1194–1217. 10.1016/j.cell.2013.05.039 23746838 10.1016/j.cell.2013.05.039 PMC3836174

[20] Franceschi C, Garagnani P, Parini P, Giuliani C, Santoro A (2018) Inflammaging: a new immune–metabolic viewpoint for age-related diseases. Nat Rev Endocrinol 14(10):576–590. 10.1038/s41574-018-0059-4 30046148 10.1038/s41574-018-0059-4

[21] Pawelec G, Goldeck D, Derhovanessian E (2014) Inflammation, ageing and chronic disease. Curr Opin Immunol 29:23–28. 10.1016/j.coi.2014.03.007 24762450 10.1016/j.coi.2014.03.007

[22] Lekamwasam R, Lekamwasam S (2020) Effects of COVID-19 pandemic on health and wellbeing of older people: a comprehensive review. Ann Geriatr Med Res 24(3):166–172. 10.4235/agmr.20.0027 32752587 10.4235/agmr.20.0027 PMC7533189

[23] Fulop T, Larbi A, Dupuis G et al (2018) Immunosenescence and inflamm-aging as two sides of the same coin: friends or foes? Front Immunol 8:1960. 10.3389/fimmu.2017.01960 29375577 10.3389/fimmu.2017.01960 PMC5767595

[24] Moderbacher CR, Ramirez SI, Dan JM et al (2020) Antigen-specific adaptive immunity to SARS-CoV-2 in acute COVID-19 and associations with age and disease severity. Cell 183(4):996–1012.e19. 10.1016/j.cell.2020.09.038 33010815 10.1016/j.cell.2020.09.038 PMC7494270

[25] Ahuja A, Sharma SK, Srivastava A, Gaur P (2022) Immunological resilience: a novel approach to understanding COVID-19 in older adults. Front Aging 3:829680. 10.3389/fragi.2022.829680

[26] Vardhana SA, Wolchok JD (2020) The many faces of the anti-COVID immune response. J Exp Med 217(6):e20200678. 10.1084/jem.20200678 32353870 10.1084/jem.20200678 PMC7191310

[27] Vabret N, Britton GJ, Gruber C et al (2020) Immunology of COVID-19: current state of the science. Immunity 52(6):910–941. 10.1016/j.immuni.2020.05.002 32505227 10.1016/j.immuni.2020.05.002 PMC7200337

[28] Cevenini E, Monti D, Franceschi C (2013) Inflamm-ageing. Curr Opin Clin Nutr Metab Care 16(1):14–20. 10.1097/MCO.0b013e32835ada13 23132168 10.1097/MCO.0b013e32835ada13

[29] Zhang Y, Ma CJ, Wang JM et al (2012) Tim-3 regulates pro- and anti-inflammatory cytokine expression in human CD14+ monocytes. J Leukoc Biol 91(2):189–196. 10.1189/jlb.1010591 21844165 10.1189/jlb.1010591 PMC3290426

[30] Bodinier M, Peronnet E, Llitjos JF et al (2024) Integrated clustering of multiple immune marker trajectories reveals different immunotypes in severely injured patients. Crit Care 28:240. 10.1186/s13054-024-04990-4 39010113 10.1186/s13054-024-04990-4 PMC11247757

[31] Anderson AC, Joller N, Kuchroo VK (2016) Lag-3, Tim-3, and TIGIT: co-inhibitory receptors with specialized functions in immune regulation. Immunity 44(5):989–1004. 10.1016/j.immuni.2016.05.001 27192565 10.1016/j.immuni.2016.05.001 PMC4942846

[32] Golden-Mason L, McMahan RH, Strong M et al (2013) Galectin-9 functionally impairs natural killer cells in humans and mice. J Virol 87(9):4835–4845. 10.1128/JVI.02605-12 23408620 10.1128/JVI.01085-12 PMC3624298

[33] Caruso C, Marcon G, Accardi G et al (2023) Role of sex and age in fatal outcomes of COVID-19: women and older centenarians are more resilient. Int J Mol Sci 24(3):2638. 10.3390/ijms24032638 36768959 10.3390/ijms24032638 PMC9916733

[34] Lowe SR, Hennein R, Feingold JH et al (2021) Are women less psychologically resilient than men? J Clin Psychiatry 83(1):21br14098. 10.4088/JCP.21br14098 34936244 10.4088/JCP.21br14098

[35] Boardman JD, Blalock CL, Button TM (2008) Sex differences in the heritability of resilience. Twin Res Hum Genet 11(1):12–27. 10.1375/twin.11.1.12 18251671 10.1375/twin.11.1.12 PMC2674367

[36] Nambi G, Abdelbasset WK, Alrawaili SM et al (2022) Comparative effectiveness study of low versus high-intensity aerobic training with resistance training in older men with post-COVID-19 sarcopenia: a randomized controlled trial. Clin Rehabil 36(2):123–136. 10.1177/02692155211036956 10.1177/02692155211036956 34344230

[37] Zamani A, Salehi I, Alahgholi-Hajibehzad M (2017) Moderate exercise enhances the production of interferon-γ and interleukin-12 in peripheral blood mononuclear cells. Immune Netw 17(3):186–191. 10.4110/in.2017.17.3.186 28680380 10.4110/in.2017.17.3.186 PMC5484649

[38] Zhao G, Zhou S, Davie A, Su Q (2012) Effects of moderate and high intensity exercise on T1/T2 balance. Exerc Immunol Rev 18:98–114 22876723

[39] Simpson RJ, Katsanis E (2020) The immunological case for staying active during the COVID-19 pandemic. Brain Behav Immun 87:6–7. 10.1016/j.bbi.2020.04.041 32311497 10.1016/j.bbi.2020.04.041 PMC7165095

[40] Yu X, Pei W, Li B et al (2025) Immunosenescence, physical exercise, and their implications in tumor immunity and immunotherapy. Int J Biol Sci 21(3):910–939. 10.7150/ijbs.100948 39897036 10.7150/ijbs.100948 PMC11781184

