# Supplementary material for: Th1/Th2 immune regulation and functional resilience in older adults following severe COVID-19: a prospective cohort study
Source: Eur Geriatr Med. 2025 Aug 31;17(2):1019–28. doi: 10.1007/s41999-025-01293-x (PMC13109217; doi:10.1007/s41999-025-01293-x)
Supplement: Supplementary file 1 — Supplementary file1 (DOCX 37 KB) [file 41999_2025_1293_MOESM1_ESM.docx]

SUPPLEMENTAL MATERIAL

eFigure 1. Workflow of enrolled patients-----------------------------------------------------------------------------1

eTable 1. Frailty assessment--------------------------------------------------------------------------------------------2

eTable 2. Antibodies used for flow cytometry and ELISA---------------------------------------------------------3

**eFigure 1. Workflow of enrolled patients**

Improved frailty status at one year of follow-up

n= 9

Baseline geriatric assessment, blood sample and frailty measurements

n=24

Frail at initial assessment

n=18

Pre-frail at initial assessment

n= 6

Improved frailty status at one year of follow-up

n= 4

Did not improve frailty status at one year of follow-up

n= 9

Did not improve frailty status at one year of follow-up

n= 2

Resilient

n= 13

Non-Resilient

n= 11

eFig 1. Workflow of enrolled patients. Twenty-four patients were recruited from a post-COVID clinic; Eighteen patients were frail at baseline, and six were pre-frail. At one year of follow-up, thirteen patients improved their frailty status, while eleven did not.

eTable 1. Frailty assessment

| Component | Measurement |
| --- | --- |
| Weight loss | Self-reported, unintentional weight loss of 5 kg or more in the previous two years, or BMI lower than 22 kg/m2. |
| Exhaustion | Affirmative response to the following question: “During the last two years, have you frequently had severe fatigue or exhaustion?” |
| Low walking speed | Affirmative response to either of the following questions: “Because of a health problem, do you experience difficultywalking one block?” and “Because of a health problem, do you have difficulty climbing flights of stairs without resting?” |
| Low physical activity | Affirmative response to the following question: “During the last two years have you exercised or done hard physical work on average at least three times a week?” |
| Weakness | Affirmative response to the following question: “Because of a health problem, do you have difficulty lifting or carrying objects over 5 kg, such as a heavy bag of groceries?” |

Fit= 0 items present

Pre-frail= 1-2 items present

Frail= 3-5 items present

eTable 2. Antibodies used for flow cytometry and ELISA

| Product | Catalog | Company |
| --- | --- | --- |
| Human TIM-3 DuoSet ELISA | DY2365 | RD |
| Human Galectin-9 DuoSet ELISA | DY2045 | RD |
| ELISA MAX™ Deluxe Set Human IFN-γ | 430104 | Biolegend |
| ELISA MAX™ Standard Set Human TNF-α | 430201 | Biolegend |
| PE/Cyanine7 anti-human CD279 (PD-1) Antibody | 329918 | Biolegend |
| PE/Cyanine5 anti-human/mouse Granzyme B Recombinant Antibody | 372226 | Biolegend |
| PE anti-human CD3 | 981004 | Biolegend |
| FITC anti-human CD8a Antibody | 301050 | Biolegend |
| APC/Cyanine7 anti-human CD273 (B7-DC, PD-L2) Antibody | 345516 | Biolegend |
| APC anti-human Perforin Antibody | 353312 | Biolegend |
| Brilliant Violet 510™ anti-human CD4 Antibody | 300546 | Biolegend |
| Brilliant Violet 421™ anti-human CD274 (B7-H1, PD-L1) Antibody | 329714 | Biolegend |
